# Natural Cinnamic Acid Derivatives: A Comprehensive Study on Structural, Anti/Pro-Oxidant, and Environmental Impacts

**DOI:** 10.3390/ma14206098

**Published:** 2021-10-15

**Authors:** Kamila Gryko, Monika Kalinowska, Piotr Ofman, Renata Choińska, Grzegorz Świderski, Renata Świsłocka, Włodzimierz Lewandowski

**Affiliations:** 1 Department of Chemistry, Biology and Biotechnology, Faculty of Civil Engineering and Environmental Sciences, Białystok University of Technology, Wiejska 45E, 15-351 Białystok, Poland; k.gryko@pb.edu.pl (K.G.); g.swiderski@pb.edu.pl (G.Ś.); r.swislocka@pb.edu.pl (R.Ś.); w-lewando@wp.pl (W.L.); 2 Department of Environmental Engineering Technology, Faculty of Civil Engineering and Environmental Sciences, Bialystok University of Technology, Wiejska 45E, 15-351 Białystok, Poland; p.ofman@pb.edu.pl; 3 Prof. Wacław Dąbrowski Institute of Agricultural and Food Biotechnology—State Research Institute, Rakowiecka 36, 02-532 Warsaw, Poland; renata.choinska@ibprs.pl

**Keywords:** cinnamic acid, phenolic acid, antioxidant, antimicrobial, biodegradation, spectroscopy

## Abstract

Cinnamic acid (CA), *p*-coumaric acid (4-hydroxycinnamic acid, 4-HCA), caffeic acid (3,4-vdihydroxycinnamic acid, 3,4-dHCA), and 3,4,5-trihydroxycinnamic acid (3,4,5-tHCA) were studied for their structural, anti-/pro-oxidant properties and biodegradability. The FT-IR, FT-Raman, UV/Vis, ^1^H and ^13^C NMR, and quantum chemical calculations in B3LYP/6-311++G** were performed to study the effect on number and position of hydroxyl group in the ring on the molecular structure of molecules. The antioxidant properties of the derivatives were examined using DPPH^●^ and HO^●^ radicals scavenging assays, ferric ion reducing antioxidant power (FRAP), cupric reducing antioxidant capacity (CUPRAC), inhibition of linoleic acid oxidation, as well as the biological antioxidant assay with *Saccharomyces cerevisiae*. Moreover, the pro-oxidant activity of compounds in Trolox oxidation assay was estimated. The effect of the derivatives on environment on the basis of increasing the carbon and nitrogen compounds transformation processes occurring in biological wastewater treatment was studied.

## 1. Introduction

**Figure 1:**
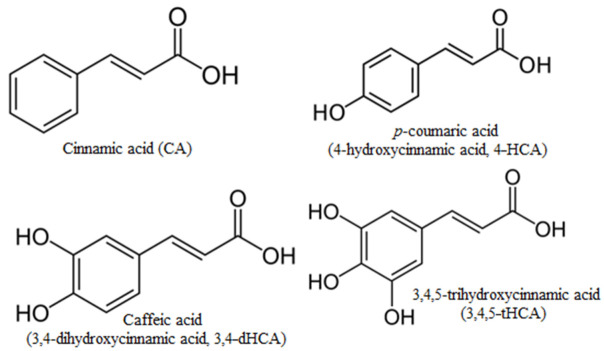
Structures of cinnamic acid and its hydroxyl derivatives.

A large group of phenolic acids belong to the natural organic matter as a product of plant degradation. The two large classes of phenolic acids found in plants are benzoic and cinnamic acids and their derivatives. The most common hydroxy derivative of cinnamic (CA) are *p*-coumaric acid (4-hydroxycinnamic acid, 4-HCA) and caffeic acid (3,4-dihydroxycinnamic acid, 3,4-dHCA), and much less common is 3,4,5-trihydroxycinnamic acid (3,4,5-tHCA) (Figure 1).

CA is a key intermediate product of the shikimic acid pathway, as a precursor of flavonoids or lignin synthesis. CA derivatives, being secondary metabolites, play important roles in plant growth, its development, reproduction, and disease resistance [1]. Due to their wide occurrence in plants, low toxicity and high biological activity, cinnamic acid derivatives are considered as food additives (antioxidants, preservatives) or pharmacologically active compounds. They can be used as initial compounds in the development of new substances, which creates the risk of their occurrence in the environment (water and soil).

Phenolic compounds are well-known antioxidants because of their high redox potential. They can act as reducing agents, scavengers of singlet oxygen, hydrogen donors, or metal ions chelators [2,3,4,5]. 3,4-dHCA is a compound that exhibits strong reducing properties, stronger than butylated hydroxytoluene (BHT) and comparable to Trolox [4]. As concluded from literature data, in both antioxidant activity assays (DPPH radical scavenging and Fe^3+^ reduction ability), the tendency of the increase in antioxidant activity was: CA < 4-HCA < ascorbic acid < 3,4-dHCA [6]. In addition, literature data showed that that 3,4-dHCA and 4-HCA were more effective antioxidants than their benzoic counterparts, i.e., protocatechuic and *p*-hydroxybenzoic acids. They react with oxidants and free radicals, forming a phenoxyl radicals, and are more stable because of the system of conjugated double bonds [7]. This way, the oxidation of other compounds is inhibited, the cell is protected from the harmful effects of reactive oxygen species (ROS), and the previous effects of their activity are reduced. Due to the presence of hydroxyl groups bounded to the aromatic ring in the molecules, phenolic compounds can be converted into semiquinones, and then into *ortho-* and *para*-quinones in reaction with free radicals [8]. The antioxidant action of monophenols is strongly enhanced by the introduction of a second hydroxyl group and a methoxy moiety in the *ortho-* position to the molecule. The stronger antioxidant power of dihydroxycinnamic acids (including 3,4-dHCA) is explained by the presence of intramolecular hydrogen bonding in *ortho*-substituted phenolic compounds [5]. As proven by the work of Un, Lee, and others, 3,4-dHCA is a good factor in oxidative stress inhibition, significantly increasing the activity of superoxide dismutase, catalase, and glutathione peroxidase, as well as reducing the level of hydrogen peroxide or thiobarbituric acid in erythrocytes and liver cells of a diabetic mouse [9]. 3,4-dHCA, ferulic, and chlorogenic acids scavenged the superoxide anion (O_2_^●−^) on a very high level, whereas 4-HCA was much less effective [10]. In the case of ferulic acid, in which the 3-hydroxyl group of 3,4-dHCA is replaced with the 3-methoxy group, a decrease in the effectiveness of the antiradical properties was observed [2].

The use of antioxidant compounds can slow down the oxidation processes and thus extend the shelf life of different food products. As there are reports of possible mutagenic and carcinogenic properties of some synthetic antioxidants [11], there is a growing demand among consumers to replace them with natural compounds which have at least as good activity as the synthetic ones. Plant-derived phenolic compounds, thanks to their antioxidant activity and other favorable biochemical properties, can be used as natural, effective antioxidants and improve the quality of food products. Moreover, CA derivatives may be applied in the prevention or treatment of many chronic civilization diseases, such as cancer, diabetes, neurodegenerative disorders, allergies, and other caused by the oxidative stress.

A better understanding of the reactions of phenolic compounds, including the hydroxyl derivatives of CA, would explain their mode of action against radicals. Furthermore, there is much evidence that phenolics, in sufficiently high concentrations, may as well act as pro-oxidants, being able to start a pro-oxidant reaction through Fe(III)–Fe(II) reduction, resulting in the formation of hydroxyl radicals [12,13]. Current data confirm that even an insignificant change in the structure (number and substitution pattern of hydroxyl groups, charge distribution, or strength of the intramolecular hydrogen bonds) can rapidly affect the anti/pro-oxidant and cytotoxic effects of chemicals [1,14,15].

Due to their antioxidant properties, hydroxycinnamic acids are the compounds with potential application as preservatives or even drugs in food, pharmaceutical, or medical industries. Moreover, they can act as potential agents in the wastewater treatment. The impact of chemical substances is a complex process that requires a number of studies. In particular, this is caused by the complexity of phenomena occurring in the environment and the relatively gradual changes that take place in it [16]. It is important to assess the effect of these compounds on environmental parameters. A more rapid method to observe that such changes may be based on intensification of carbon and nitrogen compounds transformation occurring in biological wastewater treatment. The results of such analysis cannot be directly related to the impact of selected substances on the environment; however, it allows to identify the nature of the specific compounds’ impact.

CA derivatives also exhibit other than antioxidant activities based on targeting specific enzymes, including antifungal (benzoate 4-monooxygenase inhibition) [17] or acute ischemic stroke treatment (thromboxane A2 synthase inhibition) [1]. Many compounds derived from CA show other beneficial therapeutic properties, such as anticancer [18,19], antiatherogenic [20], and antiepileptic [21].

In this paper, the properties of a series of four hydroxyl derivatives with an increasing number of hydroxyl groups in the aromatic ring were studied. The change in the number and position of −OH groups in the ring affects the electronic charge distribution over the molecules and their structural and biological (including antioxidant, lipophilic) properties. Therefore, in this work, the molecular structure of CA and the chosen CA hydroxyl derivatives was studied by means of experimental spectroscopic methods: FT-IR, FT-Raman, UV/Vis, and ^1^H and ^13^C NMR. The quantum chemical calculations in B3LYP/6-311++G** level were completed in order to obtain the optimal structure of compounds, the NBO/ESP atomics of atomic charges and ^1^H and ^13^C NMR signals, and electronic parameters calculated on the basis of the energy of HOMO and LUMO orbitals. The antioxidant properties of the compounds were examined using different spectrophotometric assays: DPPH^●^ and HO^●^ radicals scavenging, ferric ion reducing antioxidant power (FRAP) and cupric reducing antioxidant capacity (CUPRAC), inhibition of linoleic acid oxidation, as well as the biological antioxidant assay with *Saccharomyces cerevisiae*. Moreover, the pro-oxidant activity of compounds in the Trolox oxidation assay was estimated. *S. cerevisiae* was used as a model organism for the evaluation of antioxidant capacity for several reasons. It is characterized by a simple structure, very similar to human cells. Like human cells, the *S. cerevisiae* yeast cells are eukaryotic. Many human proteins have been discovered through previous studies of their yeast homologues. Moreover, *S. cerevisiae* has developed antioxidant defense systems, including both an enzymatic and non-enzymatic one [22]. The use of a cellular antioxidant activity assay with *S. cerevisiae* is advantageous over chemical methods, e.g., DPPH, FRAP, and TEAC, because it is more biologically relevant. This method takes into account the actual physiological conditions of the cell, the bioavailability of the tested compounds in the cell, and the cell’s metabolic processes. Moreover, the use of eukaryotic yeast cells in screening for antioxidant activity eliminates costly and time-consuming animal and human studies [23]. Moreover, the environmental impact of selected compounds on wastewater was also determined on the basis of increasing the carbon and nitrogen compounds transformation processes occurring in biological wastewater treatment.

## 2. Materials and Methods

### 2.1. Chemicals

All chemicals at an analytical purity were used without further purification. CA; 4-HCA; 3,4-dHCA; 3,4,5-tHCA; KBr; DPPH (2,2-diphenyl-1-picrylhydrazyl); TPTZ (2,4,6-tripyridyl-s-triazine); FeCl_3_; FeSO_4_∙7H_2_O; ammonium acetate; neocuproine (2,9-dimethyl-1,10-phenanthroline); CuCl_2_∙2H_2_O; Trolox; linoleic acid; FeCl_2_∙4H_2_O; NH_4_SCN; Tween^®^ 20; H_2_O_2_; salicylic acid; phosphate buffer (pH = 7); and horseradish peroxidase (HRP) were purchased from Sigma-Aldrich Co. (St. Louis, MO, USA). Dimethyl sulfoxide (DMSO), hydrochloric acid (35%), methanol, and ethanol (analytical grade) were purchased from Chempur (Piekary Śląskie, Poland).

### 2.2. Spectroscopic Studies

The IR spectrum of 3,4,5-tHCA was recorded with the Cary 630 FTIR Agilent Technologies spectrometer (Santa Clara, CA, USA) within the range of 400–4000 cm^−1^. Spectral resolution was 1 cm^−1^. Solid sample was measured in the KBr matrix. Raman spectrum was recorded in the range of 4000–100 cm^−1^ with an FT Raman instrument of the Perkin–Elmer System 2000 (Billerica, MA, USA). Spectral parametersof three other derivatives were compiled on the basis of literature data [24,25,26]. The UV spectra of derivatives dissolved in methanol were scanned on a DR 5000 HACH UV-Vis spectrophotometer (Loveland, CO, USA) in the wavelength range of 200–400 nm. The concentration of the derivatives was 0.01 mM. The NMR spectrum of the DMSO 3,4,5-tHCA solution was recorded with a Bruker Avance II 400 MHz unit at room temperature. Tetramethylsilane was used as an internal reference. The chemical shift values in the ^1^H and ^13^C NMR spectra of the three other derivatives were summarized based on the literature data [24,25,26,27].

### 2.3. Quantum Chemical Calculations

The optimal structures of CA; 4-HCA; 3,4-dHCA; and 3,4,5-tHCA were calculated using the B3LYP/6-311(d,p) method using the Gaussian 09 program [28]. For the structures, the electron charge distribution was calculated using the NBO [29] and Chelp [30] methods. The chemical proton ^1^HNMR and carbon ^13^CNMR shifts were calculated by the GIAO method [31] for optimal structures using the DMSO solvent. The proton and carbon chemical shift values were obtained by subtracting the proton/carbon chemical shift values from the tertramethylsilane (TMS) shift values calculated by the same method. The energy of the HOMO and LUMO orbitals as well as the energy parameters were also calculated, according to the following equations: Energy gap,

$$\mathrm{Energy}\;\mathrm{gap},\Delta E=E_{\mathrm{LUMO}}-E_{\mathrm{HOMO}};\;\mathrm{Ionizationenergy};\;I=-E_{\mathrm{HOMO}};$$

$$\mathrm{Electron}\;\mathrm{affinity},\;A=-E_{\mathrm{LUMO}};\;\mathrm{Electronegativity},\;\chi =\frac{I+A}{2};$$

$$\mathrm{Chemical}\;\mathrm{potential},\;\mu =-\frac{I+A}{2};\;\mathrm{Chemical}\;\mathrm{hardness},\;\eta =\frac{I-A}{2}\;;$$

$$\mathrm{Chemical}\;\mathrm{softness},\;S=\frac{1}{2\eta };\;\mathrm{Electrophilicity}\;\mathrm{index},\;\omega =\frac{\mu^{2}}{2\eta }$$

Based on the calculated lengths of bonds in the aromatic ring of acids, the geometric aromaticity indices were calculated. The harmonic oscillator model of aromaticity (HOMA) index differs from all other geometry-based ones by assuming another reference bond length. In this model, instead of the mean bond length a concept of the optimal bond length is applied [32].

Within the confines of the HOMA model, it is possible to obtain two components which describe different contribution to decrease in aromaticity, i.e., (a) due to bond elongation (the EN component), and (b) due to bond length alternation (the GEO component). The value of HOMA index is equal to 1 for the entire aromatic system; HOMA = 0 when structure is non-aromatic and HOMA < 0 for anti-aromatic ring. The value of the Bird’s aromaticity index (I6) describes the equation in [33]. Aj, i.e., Julg’s index equation, is described in [34]. The Bond Alternation Coefficient (BAC) index [35] determines the degree of variation in the length of adjacent bonds in the ring. The Nucleus Independent Chemical Shift (NICS) magnetic aromaticity index is defined as the negative value of the absolute chemical shift calculated at the center of the ring or in another fragment of the system [36]. The NICS value was calculated for the optimized structures of the acids using the GIAO/B3LYP/6-311++G(d,p) method.

In aromatic system, the substituent influences charge distribution via two independent effects: s-inductive and p-resonance. These effects are described by the sEDA and pEDA descriptors. The s-effect is defined as the sum of occupancies of s, px, and py valence orbitals of all the ring atoms (where xy is the ring plane). The p-effect is defined by the sum of occupancies of the pz orbitals of all the ring atoms contributing to the p-electron system [37].

The values of the sEDA and pEDA indexes were calculated using the NPA population analysis by the B3LYP/6-311++G(d,p) method for optimized structures. The reference was unsubstituted CA. The equations described in [37] were used, where σj and πj denote sums of occupancies of all atomic orbitals of the jth aromatic ring atom contributing to the valence s- and π-molecular orbitals (respectively) in the molecule, CA-cinnamic acid, and CA-(OH)x- cinnamic acid with substituted OH groups (4-HCA x = 1, 3,4-dHCA x = 2, and 3,4,5-tHCA x = 3).

### 2.4. Antioxidant Assays

#### 2.4.1. DPPH^●^ Antiradical Activity Assay

DPPH^●^ antiradical activity was measured in accordance to the DPPH^●^ assay described in [38]. DPPH^●^ was dissolved in methanol at a 60-µM concentration. First, the kinetics of DPPH^●^ radical scavenging activity was examined for each of the tested derivatives, using different concentrations. Quantities of pure substances were dissolved in methanol. In glass tubes, the given dilution of the antioxidant was added to 2 mL of the radical solution so that the final volume of the mixture was 3 mL. Control samples were prepared by adding 1 mL of methanol to 2 mL of DPPH^●^ solution. Samples were incubated in the dark for 1 h. After this time, the absorbance of the samples was measured with a NANOCOLOR VIS MACHEREY-NAGEL spectrophotometer at a wavelength of 516 nm. The antiradical activity of the acids was calculated using the formula:

$$\%I=\frac{A_{c}^{516}-A_{t}^{516}}{A_{c}^{516}}\cdot 100\%$$

where %I—percent of DPPH^●^ inhibition, $A_{c}^{516}$—absorbance of the control sample, and $A_{t}^{516}$—absorbance of the test sample.

A standard DPPH^●^ curve was made in the concentration range of 5–70 µM, on the basis of absorbance measurements every 10 min during a maximum of 6 h:

$${\mathrm{Abs}}_{516\mathrm{nm}}=11.257\times \;\left(C_{{\mathrm{DPPH}}^{●}}\right)\;+\;0.1809;{\;R}^{2}=0.9999$$

The percentage of remaining DPPH^●^ was calculated, based on the initial radical concentration in the mixture. Then, the IC_50_ values were calculated, i.e., the concentration of each tested compounds at which the initial amount of the radical was reduced by 50%. Appropriate concentration series were selected for each of the antioxidants. The assay was performed in five replicates, in three independent experiments.

#### 2.4.2. HO^●^ Antiradical Activity Assay

Antiradical activity against the HO^●^ radical was analyzed by the modification of the method described in [39]. Solutions: 8 mM of FeSO_4_∙7H_2_O, 3 mM of salicylic acid in ethanol, and 20 mM of H_2_O_2_ were prepared. Then, in five replications, the test, control, and blank samples were made. In the test sample, 0.3 mL of FeSO_4_∙7H_2_O, 1 mL of salicylic acid, and 0.25 mL of H_2_O_2_ were added to 1 mL of the derivative DMSO solution in 0.1 and 0.01 mM concentrations. In the control sample, deionized water was added instead of H_2_O_2,_ while in the blank sample, deionized water was used instead of the derivative solution. All samples were vortexed and incubated at 37 °C for 30 min. Then, 0.5 mL of deionized water was added to each of them; they were then vortexed and the absorbance was measured at λ = 510 nm, with reference to the water:DMSO = 2:1 (*v*/*v*) mixture. The percent of hydroxyl radical scavenging activity was calculated using the formula:

$$\%I=\;(1-\left(\frac{A_{t}^{510}-A_{c}^{510}}{A_{b}^{510}}\right))\cdot 100\%$$

where $A_{t}^{510}$—absorbance of the test sample, $A_{c}^{510}$—absorbance of the control sample, and $A_{b}^{510}$—absorbance of the blank sample. The determination was performed in triplicate in three independent experiments.

#### 2.4.3. Ferric Reducing Activity Assay (FRAP)

A working FRAP solution was prepared by mixing at 10:1:1 (*v/v*) ratio of three components: 300 mM of acetate buffer, 10 mM of TPTZ solution in 40 mM of HCl, and 20 mM of FeCl_3_. In order to prepare the FeSO_4_ calibration curve, a concentration series of 0.3–0.05 mM was made. Activity was determined by adding 0.4 mL of each concentration (in five replicates) to 3 mL of FRAP solution. The absorbance of the samples was measured according to the procedure described in [40] after 8 min at a wavelength of 595 nm. The activity of the tested ligands was determined at a concentration of 0.05 mM. Antioxidant activity was expressed as Fe^2+^ equivalents [µM], using the obtained calibration curve $({\mathrm{Abs}}_{595\mathrm{nm}}=33.90\times \;\left(C_{{\mathrm{FeSO}}_{4}}\right)-0.002;{\;R}^{2}=0.999$). The assay was performed in five replicates, in three independent experiments.

#### 2.4.4. Cupric Reducing Activity Assay (CUPRAC)

A working CUPRAC solution was prepared by mixing at 1:1:1: (*v*/*v*) ratio: 0.01 M of CuCl_2_, 1 M of ammonium acetate, and 7.5 mM of methanolic solution of neocuproine, in accordance to the assay described in [41]. For a calibration curve, concentrations between 0.35 mM and 0.05 mM of Trolox were prepared $({\mathrm{Abs}}_{450\mathrm{nm}}=20.38\times \;\left(C_{\mathrm{Trolox}}\right)-0.006;{\;R}^{2}=0.999$). The reducing activity was determined by adding 0.5 mL of a given concentration of antioxidant and 0.6 mL of deionized water to 3 mL of CUPRAC solution. After one hour of incubation, the absorbance was measured against a blank (methanol instead of antioxidant solution) at λ = 450 nm. The activity of the tested derivatives was examined at 0.05 mM concentration, expressed as Trolox equivalents [µM]. The assay was performed in five replicates, in three independent experiments.

#### 2.4.5. Pro-Oxidant Activity Assay

Pro-oxidant capacity was determined according to a method described in [12]. The derivatives activity was compared on the basis of their ability to oxidize Trolox. The following solutions were prepared: 0.04 µM of HRP in 0.05 M of phosphate buffer, 0.4 mM of Trolox, and 0.2 mM of H_2_O_2_. Then, pro-oxidant activity was determined for two derivatives concentrations, each in triplicate. The reaction mixture was 0.5 mL of Trolox, 0.5 mL of H_2_O_2_, 0.5 mL of HRP, then 5 or 10 µL of 1 mM derivative solution in methanol and 0.495 or 0.49 mL of deionized water (final ligand concentrations in the test tube: 2.5 or 5 µM, respectively). The reaction mixtures were mixed, then poured into quartz cuvettes, and the absorbance was measured immediately and every 20 min for an hour at λ = 272 nm, against 0.05 M of phosphate buffer. The experiment was carried out in triplicate. The formula used to calculate the increase in Trolox oxidation is as follows:

$$\%P=\frac{A_{t}^{272}-A_{c}^{272}}{A_{c}^{272}}\cdot 100\%$$

where $A_{t}^{272}$—absorbance of the test sample, $A_{c}^{272}$—absorbance of the control sample. The assay was performed in five replicates, in three independent experiments.

#### 2.4.6. Inhibition of Linoleic Acid Peroxidation Assay

For this assay, the ferric thiocyanate method was used. The procedure of [42] was slightly modified. Linoleic acid emulsions were prepared by adding to a 50 mL volumetric flask: 0.312 mL of ≥99% linoleic acid, 0.256 mL of Tween^®^ 20, and 0.05 M of phosphate buffer. Next, 1 mL of methanolic 5 mM solution of each derivative was added to 1.5 mL of linoleic acid emulsion. Emulsions were prepared in five replicates for each tested acid. At the same time, control samples were prepared, which contained 1 mL of methanol instead of the ligand solution. The glass tubes were capped and kept in a 40 °C incubator. After reaching this temperature, 0.1 mL of the solution was taken from each tube, 4.7 mL of 75% methanol, and 0.05 mL of 30% NH_4_SCN were added. Precisely after 3 min, 0.05 mL of 0.02 M FeCl_2_ 4H_2_O dissolved in 3.5% HCl was added. The absorbance was measured immediately at λ = 500 nm against 75% methanol. The measurements were performed every 24 h, each time taking 0.1 mL from the same emulsions. The activity, i.e., the percent of the peroxidation of linoleic acid inhibition, was calculated using the formula:

$$\%I=\frac{A_{c}^{500}-A_{t}^{500}}{A_{t}^{500}}\cdot 100\%$$

where $A_{c}^{500}$—absorbance of the control sample, and $A_{t}^{500}$—absorbance of the test sample. The assay was performed in five replicates in three independent experiments.

#### 2.4.7. Antioxidant Activity in *Saccharomyces Cerevisiae* Model

The *S. cerevisiae* KKP512 yeast strain used in this study was obtained from the Collection of Industrial Microorganisms (KKP), prof. W. Dąbrowski Institute of Agricultural and Food Biotechnology-State Research Institute (IAFB-SRI, Warsaw, Poland), deposited in the Department of Fermentation Technology. The yeast stock cultures were maintained at 4 °C on Petri dishes containing yeast extract peptone dextrose agar (YPD) (1% yeast extract, 1% peptone, 2% glucose, and 2% agar). For all experiments, the cells were grown in liquid Sabouraud with 2% glucose medium (1% peptone, 2% glucose) at 28 °C/160 rpm. The stock solution (0.15 M) of each studied hydroxycinnamic acids in 50% DMSO was then prepared. The work solutions (15 mM, 7.5 mM, and 3 mM) were prepared by the appropriate dilution of stock solution in Sabouraud medium prior to the experiments. The experiments were performed following modified method described by the Gao et al. [43]. Briefly, 5 mL of Sabouraud medium with hydroxycinnamic acids was inoculated with 24-h culture (the initial cell concentration was 10^5^ cells·mL^−1^), and incubated at 28 °C/160 rpm for 2 h. Then, the cells were subjected to oxidative stress induced by the addition of hydrogen peroxide (H_2_O_2_) in the concentration of 4 mM or 2 mM for 1 h. The cell viability was measured after 24 h and 48 h of incubation. Cell viability was analyzed by plating the appropriately diluted cells (1000×) in duplicate on solidified 2% Sabouraud medium. The plates were incubated at 28 °C for 48 h, and then the colonies were counted. All assays were carried out in triplicate. For comparison of mean values of particular parameters, ANOVA was performed and a post-hoc analysis (Dunetta test) was used. The significance level was 0.05. Statistica version 8.0 (Statsoft, Tulsa, OK, USA) was used.

#### 2.4.8. The Environmental Impact Assessment

##### Technological Studies

The experiment was carried out in a model system of batch reactors (SBR) operating with flocked activated sludge. The single reactor cycle was divided into four unit phases. The first phase was filling combined with anaerobic wastewater treatment lasted 1 h, the second phase was aerobic wastewater treatment lasted 5 h, while the third and fourth phases were sedimentation and decantation, which lasted 1 h each. In total, the operating cycle of a single reactor was 8 h. During the anaerobic and aerobic phase, the reactor volume was mixed with the use of a slow-speed agitator with a rotational speed of 45 rpm. During the aerobic phase, air was supplied to the individual reactors in order to maintain the dissolved oxygen concentration at 2.5 mg/dm^3^. Such an amount of dissolved oxygen helped to obtain optimal conditions for aerobic wastewater treatment. All reactors operated with an equal dry mass of active sludge of 2.0 kg/m^3^. Maintaining the same mass of active sludge in individual reactors enabled a comparison of the effects of research studies on the influence of phenolic acids on the intensity with which the transformation of carbon and nitrogen compounds occurred under anaerobic and aerobic conditions.

Model SBR reactors were fed with wastewater prepared on the basis of enriched broth (0.304 mg/dm^3^), casein peptone (0.452 mg/dm^3^), and sodium acetate (0.300 mg/dm^3^), which were a source of carbon compounds and organic nitrogen. Another component of wastewater was NH_4_Cl (0.242 mg/dm^3^), which was a source of ammonium nitrogen. Phosphorus compounds in wastewater were provided by the addition of KH_2_PO_4_ (0.032 mg/dm^3^) and K_2_HPO_4_ (0.080 mg/dm^3^). Additionally, NaCl (0.014 mg/dm^3^), CaCl_2_ (0.015 mg/dm^3^), and MgSO_4_ (0.004 mg/dm^3^), which were the source of macroelements [44], were added to wastewater. Listed substances were added to tap water filtered through an activated carbon bed to remove residual chlorine. This approach minimized the oxidation reaction of the individual components added to the wastewater with chlorine compounds. Next, 0.5 mL of CA; 4-HCA; 3,4-dHCA; and 3,4,5-tHCA at concentration of 0.01M were added to wastewater. It should be noted that all of the studied acids were dissolved in methanol. Therefore, in order to preserve the same amount of carbon compounds, 0.5 mL of methanol of HPLC purity was added to the control reactors. In addition, methanol is also used as an external carbon source in wastewater treatment processes [45], and its addition to wastewater results in increased efficiency of the denitrification process [46].

##### Analytical Methodology

Wastewater samples for analytical studies were collected after the filling, anaerobic, and aerobic phases. The chemical oxygen demand (COD), as well as the N-NH_4_^+^ (ammonia nitrogen) and N-NO_3_^−^ (nitrate (V) nitrogen) concentrations observed after the filling phase, were the initial point to assess the impact of individual phenolic acid on the carbon and nitrogen transformations, both under anaerobic and aerobic conditions. Samples collected after the anaerobic phase helped to determine the effect of the studied CA derivatives on the intensity of degradation of carbon compounds under anaerobic conditions and on the course of ammonification and denitrification processes [47]. Moreover, analysis of samples after the aerobic phase revealed the influence of the studied compounds on the degradation of carbon compounds under aerobic conditions and on the course of nitrification process [48]. The analysis of COD, N-NH_4_, and N-NO_3_ concentrations was performed with the use of UV-VIS spectrophotometer Pharo 300 by Merck. The analysis was performed using dedicated reagents and the procedure specified by the manufacturer. These procedures are compliant with American Public Health Association (APHA) [49]. However, due to the complexity of phenomena occurring during biological wastewater treatment, the experiment was performed in three series to uniform obtained results. The obtained results of statistical analysis helped to indicate statistically significant differences at the level of α = 0.05. The key aspect of the analysis of variance carried out with the Tukey’s HSD test is the fulfillment of two criteria, which includes normal distribution and uniformity of variance [50]. Therefore, all variables included in the analysis were characterized by normal distribution according to the Shapiro–Wilk test and homogeneity of variance according to Bartlett’s test results.

## 3. Results

### 3.1. Spectroscopic Studies

#### 3.1.1. FT-IR and FT-Raman Spectra

The wavenumbers, intensities, and assignments of the bands occurring in the FT-IR and FT-Raman spectra of CA; 4-HCA; 3,4-dHCA; and 3,4,5-tHCA are presented in Table 1 and illustrated in Figure S1. The bands were numbered along with the notation used by Varsányi [51]. In the IR spectra of hydroxyl acids, there were bands derived from the stretching vibrations of the hydroxyl group (ν(OH)_ar_). In the 4-HCA spectrum, there was a strong band at 3383 cm^−1^, in the 3,4-dHCA and 3,4,5-tHCA spectra, and two bands at 3431, 3231 cm^−1^ and 3515, 3403 cm^−1^, respectively, were present. A characteristic band occurring in the spectra of all tested compounds was a very intense band from stretching vibrations of the carbonyl group (ν(C=O)). It occurred at 1685 cm^−1^ (IR)-CA, 1672 cm^−1^ (IR)-4-HCA, 1645 cm^−1^ (IR)-3,4-dHCA, and 1668 cm^−1^ (IR)-3,4,5-tHCA. There was also an intense band in the spectra coming from the ν(C-OH) stretching vibration located at: 1268 cm^−1^ (IR)-CA, 1283 cm^−1^ (IR), 1282 cm^−1^ (R)-4-HCA, 1279 cm^−1^ (IR), 1288 cm^−1^ (R)-3,4-dHCA, and 1295 cm^−1^ (IR)-3,4,5-tHCA. A characteristic band resulting from the stretching vibration of the double bond ν(C=C) of the aliphatic chain occurred at 1629 cm^−1^ (IR), 1638 cm^−1^ (R)-CA, 1628 cm^−1^ (IR), 1636 cm^−1^ (R)-4--HCA, 1620 cm^−1^ (IR), 1615 cm^−1^ (R)-3,4-dHCA, 1621 cm^−1^ (IR), and 1610 cm^−1^ (R)-3,4,5-tHCA. These bands, as well as the bands derived from the vibrations of the aromatic ring, such as 9a, 12, 5, and 10a, were systematically shifted towards lower wavenumber values in the series CA-4-HCA-3,4--dHCA (without 3,4,5-tHCA). This suggests that, with the increase in the number of hydroxyl substituents in the ring, the perturbation of the electronic system of ligands increase as well. The decrease in aromaticity in this series is also confirmed by the decreasing values of the aromaticity indices (i.e., HOMA, I6, Aj, BAC) obtained from the calculated data for these ligands (discussed below).

#### 3.1.2. UV Spectra

**Figure 2:**
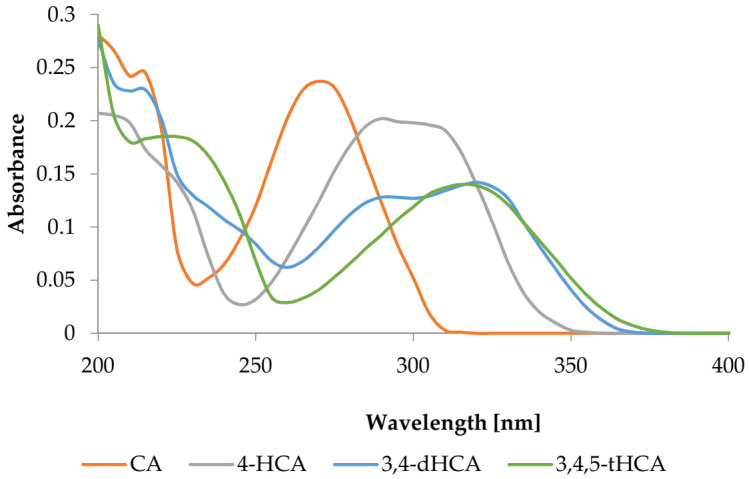
UV spectra of CA; 4-HCA; 3,4-dHCA; and 3,4,5-tHCA in methanol.

The UV spectra of CA; 4-HCA; 3,4-dHCA; and 3,4,5-tHCA are shown in Figure 2. The obtained wavelengths of maximum absorbance are collected in Table 2. The maximum of these bands were shifted toward higher wavelengths in the order: CA; 4-HCA; 3,4-dHCA; 3,4,5-tHCA. Introduction of the hydroxyl group (containing lone electron pair) to the aromatic ring caused a batochromic shift in the position of the absorption bands, generally with a hypochromic effect.

##### H and ^13^C NMR Spectra

**Figure 3:**
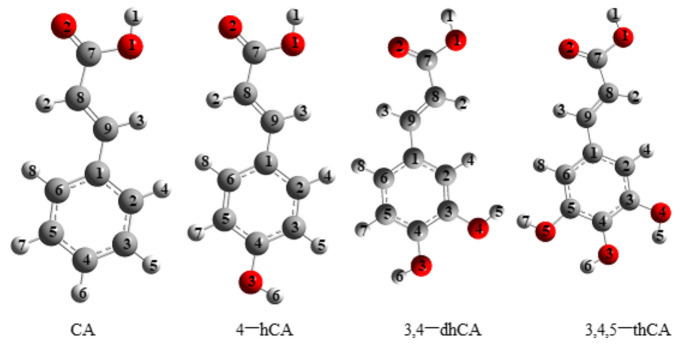
Atom numbering of cinnamic acid and its derivatives (structures optimized in B3LYP/6-311**(d,p) level).

The values of the chemical shifts from the experimental and theoretical ^1^H and ^13^C NMR spectra of CA; 4-HCA; 3,4-dHCA; and 3,4,5-tHCA were gathered in Table 3. The atom numbering is shown in Figure 3. As in the case of the IR spectra, in the ^1^H and ^13^C NMR spectra, a clear movement of the chemical shifts was observed in the series CA → 4-HCA → 3,4-dHCA → 3,4,5-tHCA. Substitution of the hydroxyl group/groups caused significant changes in the distribution of the electronic charge in molecules, especially in the aromatic ring, expressed by chemical shifts of the signals from the ^13^C NMR spectra. A systematic/regular decrease in electronic charge density around the C9 atom and an increase around C1 and C8 atoms was observed in the series CA → 4-HCA → 3,4-dHCA → 3,4,5-tHCA. The value of the chemical shift of the H6 atom from the −OH group decrease in the series 4-HCA → 3,4-dHCA → 3,4,5-tHCA. This indicates an increase in the charge density around the H6 atom along with the substitution of consequent group in the aromatic ring. Minor regular differences were observed in the chemical shifts of the remaining protons. The electron density around the atoms H2, H3, H4, H8 regularly increased in the series CA → 4-HCA → 3,4-dHCA → 3,4,5-tHCA.

### 3.2. Quantum Chemical Calculations at B3LYP/6-311**(d,p) Level

The distances between atoms in CA; 4-HCA; 3,4-dHCA; and 3,4,5-tHCA and the angles between bonds are calculated and presented in Table S1. Their energy values are calculated and presented in Table 4, in accordance to atoms numbering presented in Figure 3.

**Figure 4:**
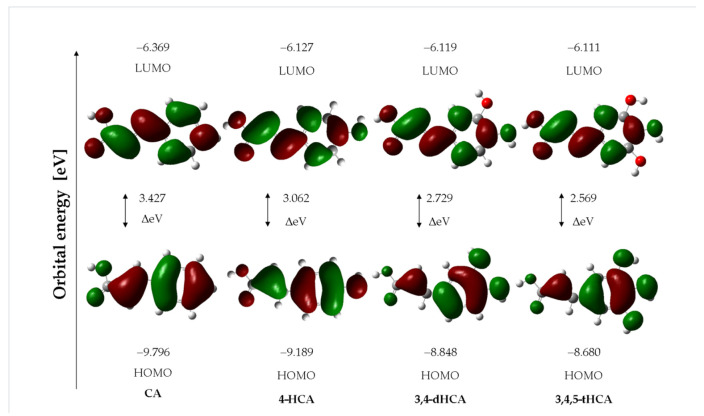
The HOMO/LUMO electron densities in molecules of studied compounds.

For the optimal structures of monomers, the geometrical aromaticity indices (HOMA, GEO, EN, I6, BAC, Aj), sEDA and pEDA indices, energy of HOMO and LUMO orbitals (Figure 4), atomic charge distribution (NBO, ESP), and theoretical signals from ^1^H and ^13^C NMR spectra were calculated at the B3LYP/6-311**G(d,p) level. These parameters are closely related to the reactivity of molecules. The substitution of one hydroxyl group in the aromatic ring of CA caused a decrease in the aromaticity of the π-electron system (Table 4) which is shown by the decrease in the values of calculated aromaticity indices. So, the aromaticity of 4-HCA is lower than CA, and substitution of the following −OH group in 3,4--dHCA caused further decrease in the aromaticity of derivatives. This was due to an increase in variation in bond lengths and increase in the asymmetrization of the electronic charge distribution in the aromatic rings of derivatives. On the contrary, the presence of three −OH substituents in the ring in 3,4,5-tHCA caused symmetrization of the electronic charge distribution, alignment of the bond lengths in the aromatic ring, and, consequently, an increase in ring aromaticity in the series of acids. Therefore, the aromaticity indices obtained for 3,4,5-tHCA were higher than those ones calculated for mono- and disubstituted CA. The magnetic aromaticity index NICS showed a decrease in the aromaticity of molecules in the order: CA > 4-HCA > 3,4-dHCA > 3,4,5-tHCA.

**Figure 5:**
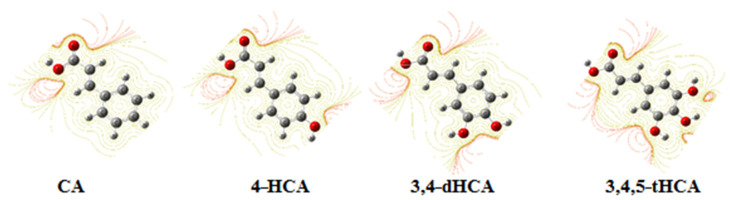
Density electronic charges (ESP) for CA; 4-HCA; 3,4-dHCA; and 3,4,5-tHCA acid calculated in CHelp/B3LYP/6-311++G(d,p).

The NBO and ESP (Table S2) analysis showed that electronic charge distribution in −COOH group of derivatives did not change significantly after substitution of −OH in the ring, whereas the electronic charge distribution in the ring is affected by the number and position of −OH groups in the ring, especially around the substituted carbon atoms. The electronic charge density around these carbon atoms significantly decreased as a result of shifting the electron charge towards the electronegative oxygen of the substituted hydroxyl group. The change in the ESP potential is illustrated in Figure 5. With the substitution of successive hydroxyl groups in the series of 4-HCA (one −OH group) → 3,4-dHCA (two −OH groups) → 3,4,5-tHCA (three −OH groups), contour lines defining the ESP potential near oxygen atoms of hydroxyl groups were densified. This influences the changes in the distribution of the electronic charge in the aromatic ring, and thus the aromaticity and reactivity of the π-electron system of the ligands.

On the basis of the results of NBO analysis, the sEDA (index describing the inductive effect) and pEDA (parameter describing the mesomeric effect) were calculated and gathered in Table S2. The pEDA parameter relates to the π-electron substituent effect (the resonance effect), while the sEDA parameter concerns the σ-electron substituent effect (the inductive effect) related to electronegativity. The pEDA index has a negative value for π-electron-accepting substituents (such as −OH group) and a positive value for π-electron-donating substituents. The lower the value of the pEDA index, the stronger the effect. In the case of the tested acids, we observed an increase in the mesomeric effect with the increase in the number of substituted hydroxyl groups. For σ-electron-donating substituents, the sEDA has a positive value, and negative values for σ-electron withdrawing (−OH) substituents. The strongest inductive effect (sEDA index) was observed in the case of the substitution of two hydroxyl groups in the CA molecule. Substitution of the third hydroxyl group reduces this effect.

The energy of HOMO and LUMO orbitals is an important parameter in predicting the electron charge transfer in a molecule, chemical reactivity/bioactivity, and compound stability [52,53]. The shapes of the LUMO and HOMO molecular orbitals of the studies acids are shown in Figure 4. The values of the energy of the HOMO and LUMO orbitals and the difference between the energies of the HOMO-LUMO (GAP) levels of the compounds are presented in Table 4. With the increase in the number of substituted hydroxyl groups in the aromatic ring of CA derivative, a decrease in the GAP value was observed, which indicates a decrease in the kinetic stability of these molecules and an increase in their reactivity. This trend follows the changes in the aromaticity of the compounds. A molecule of CA with the most stable aromatic ring is characterized by a lower reactivity, according to the GAP value, compared to 4-HCA; 3,4-dHCA; and 3,4,5-tHCA. Other general reactivity descriptors [52,53], such as ionization potential (I), electron affinity (A), electronegativity (χ), chemical hardness (η), softness (*S*), and electrophilicity index (ω), calculated on the basis of the values of the energy of the orbitals, were gathered in Table 4. These data showed that the chemical hardness decreases in the series: CA > 4-HCA > 3,4-dHCA > 3,4,5-tHCA. The electrophilicity index increases in the series: CA < 4-HCA < 3,4-dHCA < 3,4,5-tHCA, which indicates that trihydroxycinnamic acid had the highest electrophilic power and CA had the lowest one.

### 3.3. Antioxidant Properties

In this study, a number of methods for testing antioxidant activity were used, which are based on different mechanisms of action. This is an appropriate approach as there is not one comprehensive technique for such evaluation. Different methods of antioxidant activity testing can produce significantly different results (Huang et al., 2005).

#### 3.3.1. DPPH^●^ and HO^●^ Antiradical Activity Assay

**Figure 6:**
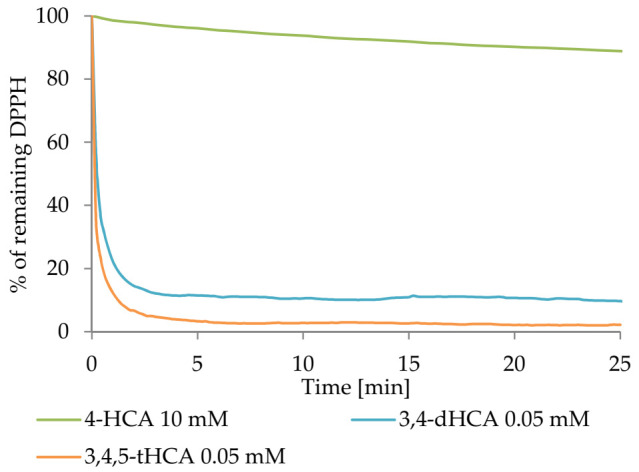
The DPPH^●^ radical scavenging kinetics of chosen compounds.

Figure 6 shows the kinetics curve of reaction between studied compounds and DPPH^●^ radical. The data for CA were excluded, as it still did not show any significant antiradical properties at the high concentration (10 mM). After 6 h of measurements, about 61% of the initial radical concentration remained as a result of the reaction with 10 mM 4-HCA. After 7–8 min, in the tests with 3,4-dHCA and 3,4,5-tHCA at a 0.05mM concentration, approximately 11.5 and 3.3% of the initial concentration of the radical remained, respectively. For 4-HCA, after the same time, about 89% of the radical remained in the sample.

**Figure 7:**
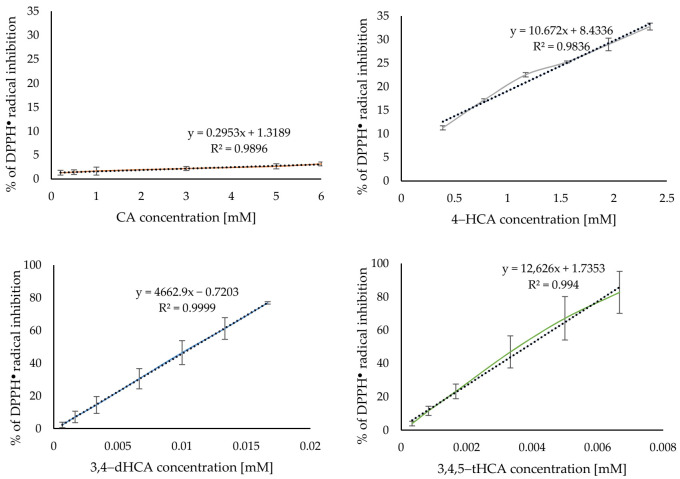
Antiradical activity of CA and its derivatives based on the DPPH^●^ radical assay.

Figure 7 shows the obtained curves of the DPPH^●^ radical inhibition caused by the applied antioxidant at increasing concentration. The use of a 6mM CA only inhibited about 3% of the initial concentration of radical, while even 1000 times lower concentration of 3,4,5-tHCA scavenged almost 80% of the initial concentration of radical. Table S3 summarized the calculated IC_50_ values. On their basis, it was concluded that the antiradical activity of CA and its derivatives increased with the increasing number of −OH group in the aromatic ring. This tendency was similar to that obtained by Jitareanu and Tataringa [6].

**Figure 8:**
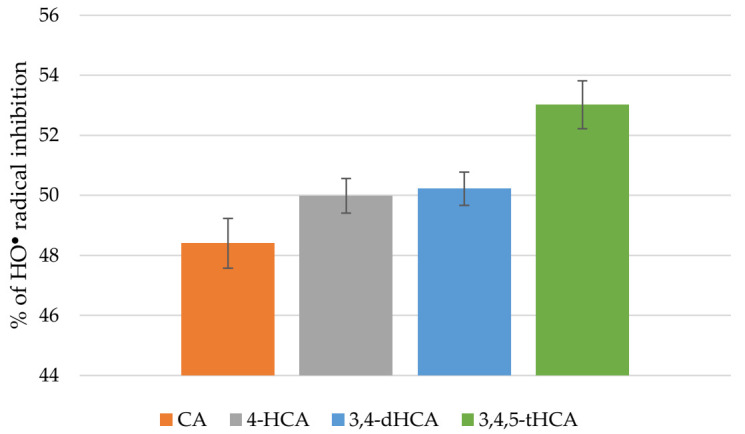
Antiradical activity of CA and its derivatives based on the HO^●^ radical assay.

CA and its derivatives in a similar way scavenged the HO^•^ radical (Figure 8). The results showed that % of inhibition of HO^•^ radical was in the range: 48.40 ± 0.83%–53.02 ± 0.80%.

#### 3.3.2. Reducing Activity Assays

**Figure 9:**
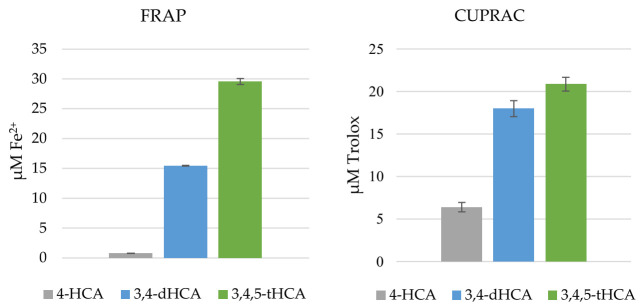
Reducing activity of CA and its derivatives, evaluated by FRAP and CUPRAC assays (the concentration of studied compounds was 0.05 mM).

The results of the FRAP and CUPRAC assays are presented in Figure 9. CA did not show reducing properties in the tested concentration in both assays. The ability to reduce Fe(III) and Cu(II) ions increased in the following order: CA < 4-HCA (0.74 ± 0.06 µM_Fe2+_; 6.40 ± 0.55 µM_Trolox_) < 3,4-dHCA (15.45 ± 0.49 µM_Fe2+_; 17.99 ± 0.94 µM_Trolox_) < 3,4,5-tHCA (29.56 ± 2.02 µM_Fe2+_; 20.86 ± 0.81 µM_Trolox_), at the concentration of studied compounds. The differences in the reduction in Cu(II) ions by 3,4-dHCA and 3,4,5-tHCA were much smaller than in the case of Fe(III) ions.

#### 3.3.3. Pro-Oxidant Activity Assay

**Figure 10:**
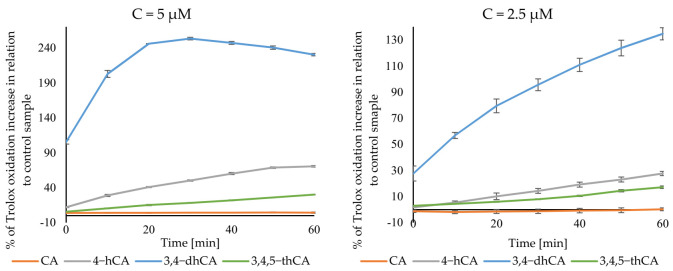
Pro-oxidant activity of CA and its derivatives.

The curves obtained in a pro-oxidant activity analysis are shown in Figure 10. As can be seen, the results of this assay do not show the same trend of increase activity of tested compounds with the increasing number of hydroxyl substituent in the aromatic ring. 3,4-dHCA was definitely the most active this time, reaching the maximum activity of 134.78 ± 4.65% at the concentration of 2.5 µM after 60min. Of the measurements, while at the concentration of 5 µM after 40 min, it was 247.24 ± 2.04%. 4-HCA was second in the terms of activity, giving a maximum of 27.63 ± 1.58 and 70.57 ± 1.18% increase in Trolox oxidation. 3,4,5-tHCA was third, giving 17.15 ± 0.96% and 30.18 ± 0.48%. CA had the weakest activity and achieved a maximum of 0.20 ± 1.09 and 4.35 ± 0.91% increase in Trolox oxidation for 2.5 and 5 µM concentrations, respectively.

#### 3.3.4. Inhibition of Linoleic Acid Peroxidation Assay

**Figure 11:**
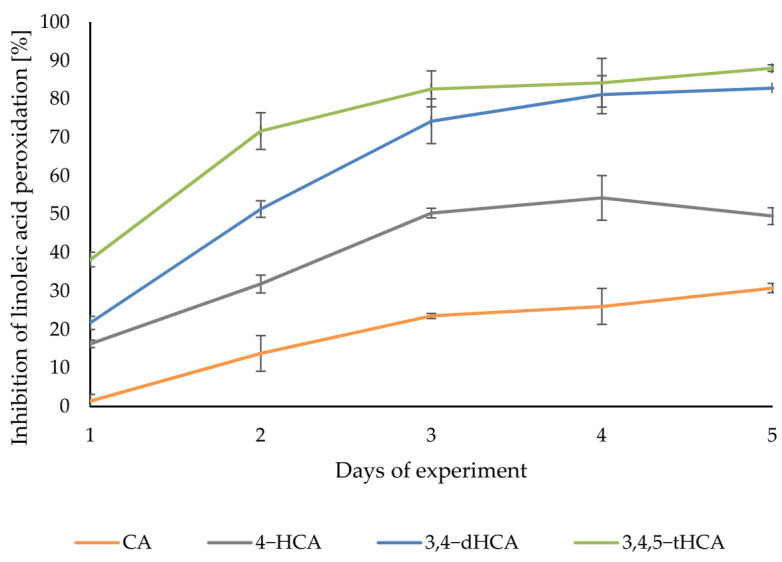
The degree of linoleic acid peroxidation inhibition by CA; 4-HCA; 3,4-dHCA; and 3,4,5-tHCA.

Figure 11 showed the curves of the degree of inhibition of linoleic acid peroxidation during incubation with the antioxidant. In the case derivatives of CA, the inhibition of linoleic acid oxidation was observed already in the first day of measurements. In the following days, the percent of inhibition of lipid peroxidation increased for each of the compounds tested. The highest level of inhibition was obtained for 3,4,5-tHCA, with the highest value of 88.01 ± 0.88% in the last day of measurements. 3,4-dHCA was slightly less active, reaching a maximum of 82.77 ± 0.12%. 4-HCA finally inhibited lipid peroxidation in 49.49 ± 2.18%. CA also prevented the oxidation of linoleic acid the least, reaching the maximum value of 30.76 ± 1.22%.

#### 3.3.5. Antioxidant Activity in *Saccharomyces Cerevisiae* Model

Antioxidant activity of CA and its derivatives against oxidative damage of *S. cerevisiae* KKP512 yeast cells induced by hydrogen peroxide was tested in culture media during 24 h and 48 h of incubation. *S. cerevisiae* cells are sensitive to oxidative stress caused by the addition of hydrogen peroxide, which is a source of reactive oxygen species [17]. The assessment of the ability of studied acids to protect *S. cerevisiae* KKP512 cells was based on the determination of viability and growth of yeast after treatment with 4-mM and 2-mM hydrogen peroxide solutions. An effect of compounds alone on the growth of *S. cerevisiae* KKP512 was also assessed. The CA and its derivatives (DMSO solutions) were used at the final concentration of 15 mM, 7.5 mM, and 3 mM. The obtained results are summarized in Table 5. The used concentration of DMSO did not show any toxic effect on yeast. The inhibitory effect on *S. cerevisiae* KKP512 during the whole incubation time was observed only for CA, independent on the concentration used. A similar effect of CA on the growth suppression of yeast and fungi was reported by other authors [17,49,50]. In the case with 4-HCA, its toxic effect on the yeast cells was observed only at the highest concentration i.e., 15 mM. For lower concentrations of the acid, *S. cerevisiae* KKP512 was able to grow but the number of colonies was significantly lower than that of control (*S. cerevisiae* KKP512 without acids). 3,4-dHCA and 3,4,5-tHCA, having more hydroxyl groups in the aromatic ring, were non-toxic to *S. cerevisiae* KKP512 in the studied concentration range. The addition of 4 mM of H_2_O_2_ suppressed the growth of pretreated hydroxycinnamic acids and untreated *S. cerevisiae* KKP512 cells after 24 h of incubation. The number of viable cells decreased considerably. However, upon further incubation, during the next 24 h, the number of viable cells in pretreated and control samples increased about two log CFU·mL^−1^ and did not differ significantly, except with 7.5 mM of 4-HCA. The obtained results clearly indicate that, despite initial growth inhibition induced by hydrogen peroxide, the yeast cells survived and were able to grow. However, no protective effect of acids on the yeast cells was observed compared to the control.

A different behavior was observed upon the addition of 2 mM of hydrogen peroxide to the pretreated and control samples. *S.cerevisiae* KKP512 pretreated with 15 mM and 7.5 mM 3,4-dHCA and 4-HCA characterized a significantly higher number of cells than control, indicating their stimulatory effect. For 3 mM solutions of 4-HCA; 3,4-dHCA; 3,4,5-tHCA; and 7.5 mM of 4-HCA, the number of cells was lower and comparable with the control. After the next 24 h of incubation, the number of viable cells increased, especially with 3 mM solutions of all tested acids and control. Only in the case with 7.5 mM 4-HCA and 3,4-dHCA, the number of viable cells remained unchanged during the whole incubation period. The obtained results showed that the observed differences in the cell growth of *S.cerevisiae* KKP512 was both structure- and dose-dependent. Several studies have related the structure–inhibition relationships of CA and its derivatives against microorganisms, especially fungi, to their inhibitory activity towards target enzymes [54,55,56]. The reported antifungal effectiveness of various natural and synthetic cinnamic acid derivatives differed depending on the functionality and position of the substituents as well as on the used microorganisms. Our results indicate that the presence of one and more substituted hydroxyl groups in the phenyl ring of CA led to a decrease in inhibitory activity towards yeast. This finding supported the observation made by Podobnik et al. [56] concerning the effect of electron-withdrawing and electron-donating groups on antifungal properties. Further investigation, including computer modeling, is needed to evaluate the binding activity of these derivatives on the specific enzymes.

### 3.4. The Environmental Impact Assessment

Uniform concentration of carbon and nitrogen forms in wastewater in after the filling phase was a critical aspect of laboratory analysis. Therefore, in all reactors, COD after the filling phase was, on average, equal to 717.00 mg·dm^−3^, while N-NH_4_^+^ and N-NO_3_^−^ concentrations were, on average, equal to 20.80 mg·dm^−3^ and 7.27 mg·dm^−3^, respectively.

**Figure 12:**
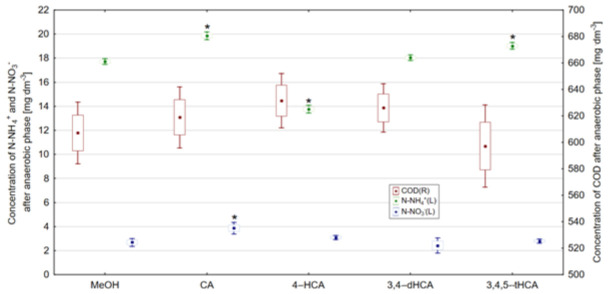
Concentration of N-NH_4_^+^, N-NO_3_^−^ and COD after anaerobic phase, *—result differing with statistical significance at α = 0.05 in accordance with the control reactor (point—arithmetic mean, box—standard error, line—standard deviation).

Degradation of carbon compounds expressed by COD was observed in all reactors regardless of the addition of phenolic acid as a result of biological transformations in anaerobic phase (Figure 12). After comparison of the obtained results with Tukey’s HSD test, no statistically significant differences were observed between COD values in individual reactors. This observation may result from the fact that, in all reactors, biodegradation of carbon compounds was observed and the process efficiency did not differ in individual samples by more than 17%.

Another process that took place during the anaerobic wastewater treatment was ammonification (Figure 12). It should be noted that the source of organic nitrogen in prepared wastewater was enriched broth and peptone K. Changes in N-NH_4_^+^ concentration during anaerobic wastewater treatment can be used to directly assess the ammonification intensity [57]. In the reactor with addition of 4-HCA, a concentration of N-NH_4_^+^ was lower than that observed in the control reactor (17.71 mg·dm^−3^). This observation may indicate that 4-HCA inhibits the ammonification process. In turn, in reactors operating with the addition of CA and 3,4,5-tHCA, the ammonification process took place with higher intensity in comparison to the control reactor, and the observed N-NH_4_^+^ concentrations were equal to 19.86 and 19.02 mg·dm^−3^. The described dependencies were reflected in Tukey’s test results. Concentrations of N-NH_4_^+^ in reactors with studied phenolic acids differed with statistical significance in comparison to the control reactor. Statistically significant differences were not observed between N-NH_4_^+^ concentrations in the control reactor and the reactor with the addition of 3,4-dHCA. This observation resulted from similar concentration of this form of N-NH_4_^+^ in those reactors.

The last of the processes occurring during anaerobic wastewater treatment was denitrification, which consists in the decomposition of aerobic nitrogen forms into gaseous nitrogen [58,59]. Statistically significant differences in N-NO_3_^−^ concentrations were observed between the reactor with the addition of CA. Observed differences were related to lower intensities of a denitrification course in the reactor with the addition of CA, which resulted in a higher concentration of N-NO_3_^−^ after the anaerobic phase (3.87 mg·dm^−3^). In case of other studied phenolic acids, the concentration of N-NO_3_^−^ was similar to that observed in the control reactor.

**Figure 13:**
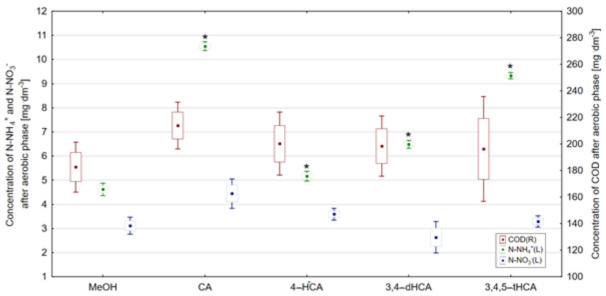
Concentration of N-NH_4_^+^, N-NO_3_^−^, and COD after aerobic phase, *—result differing with statistical significance at α = 0.05 in accordance with the control reactor (point—arithmetic mean, box—standard error, line—standard deviation).

Under aerobic conditions, the most effective degradation of carbon compounds expressed as COD was observed in the control reactor (Figure 13). It should be emphasized that the addition of studied acids did not significantly affect the COD degradation under aerobic conditions. This observation is confirmed by the results of Tukey’s NIR test, where no statistically significant differences (α = 0.05) were observed in COD concentration after the aerobic phase.

Nitrification, which results in the oxidation of ammonium nitrogen to nitrate (V) nitrogen [48], in all reactors operating with the addition of studied acids, occurred with lower intensity compared to the changes observed in the control reactor. In the reactor operating with the addition of CA, the concentration of N-NH_4_^+^ equaled to 10.55 mg·dm^−3^, and the addition of 3,4,5-tHCA resulted in the concentration of N-NH_4_^+^ after aerobic phase equal to 9.33 mg·dm^−3^. The addition of 4-HCA had the smallest influence on the course of nitrification and, in its presence, the concentration of N-NH_4_^+^ was 5.17 mg·dm^−3^. The addition of 3,4-dHCA helped to obtain the concentration of N-NH_4_^+^ equal to 6.49 mg·dm^−3^. Differences in N-NH_4_^+^ concentration were reflected in Tukey’s NIR test results, where particular concentrations of N-NH_4_^+^ were statistically significantly different in comparison with the control reactor. On the other hand, N-NO_3_^−^ concentrations observed after the aerobic phase are a consequence of the degradation of this form of nitrogen at the anaerobic wastewater treatment stage. Therefore, no statistically significant differences in concentrations of this form of nitrogen were observed in any of the reactors.

## 4. Conclusions

The results of several chemical and biological antioxidant assays based on different antioxidant mechanisms showed that the antioxidant properties of CA and its hydroxyl derivatives increase in the following order: CA < 4-HCA < 3,4-dHCA < 3,4,5-tHCA (Table S3). It means that the presence of the next hydroxyl group in the ring increases the antioxidant activity in these compounds. However, in the pro-oxidative assay, 4-HCA showed greater activity than 3,4,5-tHCA, and the strongest oxidation of Trolox was caused by 3,4-dHCA. Pro-oxidant activity of the compounds was not dependent on the presence of additional hydroxyl group in the aromatic ring. Their oxidizing capacity pathway is based on transient-free radicals which are formed in the reaction with Trolox and H_2_O_2_/HRP. The question is if the phenolic radical is able to cause an oxidation or generate a final unreactive product, as expected for a typical antioxidant [60].

The high antioxidant property of studied compounds gives a chance for broad application of these chemicals. Therefore, studies concerning their impact on environment are of great importance. According to the results obtained in this work, under anaerobic conditions, it was observed that the presence of CA, 4-HCA, or 3,4-dHCA in the effluent slightly inhibited the degradation of carbon compounds expressed as COD in comparison to the control reactor. The addition of 3,4,5-tHCA, on the other hand, had similar effects on COD biodegradation as in the control reactor. That observation indicated that 3,4,5-tHCA did not affect the carbon biodegradation under anaerobic conditions. In contrast, under aerobic conditions, the addition of the individual studied compounds resulted in inhibition of COD biodegradation. In case of denitrification, inhibitory effects were observed in the reactor with the addition of CA. In other reactors, denitrification occurred with similar efficiency. The nitrification process took place with the greatest intensity in the control reactor. Therefore, the addition of each of the studied compounds had an inhibitory effect on N-NH_4_^+^ transformation under aerobic conditions.

The biological properties in the study depend on their structure which was characterized by different spectroscopic and theoretical parameters. The differences in discussed spectral and theoretical parameters did not change regularly after substitution consecutive −OH group in the aromatic ring. This was due to an increase in the differentiation in bond lengths and an increase in the asymmetrization of the electronic charge in the molecule after substituting the first and then the second −OH group. Subsequently, the addition of the third −OH substituent in the ring caused symmetrization of the electronic charge distribution, alignment of the bond lengths in the aromatic ring, and, consequently, a slight increase in the aromaticity of 3,4,5-tHCA comparing with 4-HCA and 3,4-dHCA. With the increase in the number of substituted hydroxyl groups in the aromatic ring of CA, a decrease in the GAP value (a difference between the energy of HOMO and LUMO orbitals) was observed, which indicates a decrease in the kinetic stability of these molecules and an increase in their reactivity. A molecule of CA with the most stable aromatic ring is characterized by the lowest antioxidant activity. The spectroscopic and theoretical parameters may be good predictors of antioxidant activity of chemicals.

## Figures and Tables

**Table 1:** The wavenumbers (cm^−1^) and assignments of bands occurring in FT-IR and FT-Raman spectra of CA; 4-HCA; 3,4-dHCA; and 3,4,5-tHCA.

		CA (Kalinowska et al., 2007)		4-HCA (Świsłocka et al., 2012)		3,4-dHCA (Świsłocka, 2013)		3,4,5-tHCA	
Assignment	No. [51]	IR	Raman	IR	Raman	IR	Raman	IR	Raman
ν ^a^(OH)_ar_		-	-	3383 s ^b^	-	3431 s 3231 s	-	3515 s 3403 vs	-
ν(CH)	20a	3087 w	-	3080 w	3069 w	2926 w	-	3071 w	-
ν(CH) + ν(CH)_C=C_	20b	2992 w	-	3026 w	3025 vw	3026 w	-	-	-
ν(CH) + ν(CH)_C=C_	7b	-	-	2963 w	-	-	-	2961 w	-
ν(C=O)		1685 vs	-	1672 vs	-	1645 vs	1642 s	1668 s	1640 s
ν(C=C)		1629 vs	1638 vs	1628 s	1636 m	1620 vs	1615 vs	1621 vs	1610 vs
ν(CC)	8a	1600 sh	1600 s	1601 vs	1606 vs	1599 s	1596 m	1597 vs	-
ν(CC)	8b	1577 m	-	1591 s	1593 m	1530 m	1533 w	1547 m	-
ν(CC)	19a	1495 m	1496 vw	1512 s	1519 w	1525 vw	1524 m	1538 m	-
ν(CC)	19b	1449 s	1444 vw	1449 vs	1448 w	1450 vs	1453 vw	1454 m	1443 m
ν(CC) + β(OH)	14	1334 m	1329 vw	1379 m	-	1352 m	1354 w	1364 w	1372 w
β(CH)_C=C_ + β(CH)	3	1389 w	-	1314 s	1306 w	-	-	1318 s	1309 w
ν(C-OH)		1286 s	-	1283 m	1282 w	1279 vs	1288 sh	1295 vs	-
β(OH)		-	-	1244 vs	1260 m	-	1108 w	1224 s	1231 m
β(CH)	13	1206 m	-	1215 vs	1213 m	-	-	1191 m	1190 w
β(CH)	9a	1176 m	1179 m	1173 s	1172 s	-	-	1161 m	1157 m
β(CH)	18b	1073 w	-	1105 m	-	1121 s	1125 w	1005 w	1007 w
β(CH)	18a	1027 w	1027 w	1013 w	-	1175 m	1187 m	1031 s	1037 w
α(CCC)	12	999 w	1002 m	799 m	800 w	799 m	780 vw	803 w	808 w
γ(CH)	17a	-	-	941 m	952 vw	935 w	957 vw	984 m	986 w
γ(CH)_C=C_ + γ(CH)	17b	-	-	833 s	837 w	974 m	975 w	-	-
γ(CH)	5	-	-	-	-	849 m	853 w	-	-
γ(CH)	10a	875 w	876 vw	860 w	864 w	800 w	804 w	868 w	870 w
γ(CH) + γ(HCCO) + γ(OCOC)	11	769 s	771 vw	-	-	-	-	831 m	831 w
γ(CO)		-	-	920 m	-	698 m	-	732 w	719 vw
φ(CC)	4	711 s	714 vw	646 w	645 vw	696 w	688 vw	673 vw	-
α(CCC)	6b	683 m	681 vw	-	-	552 w	460 w	-	-
α(CCC)	6a	-	-	-	-	602 w	606 vw	-	631 w
φ(CC)	16a	-	-	453 w	-	-	-	-	-
φ(CC)	16b	483 m	-	517 m	516 vw	457 w	447 w	-	-
β(CH)	9b	-	-	430 vw	421 vw	-	-	-	443 vw

^a^ „ν”—stretching vibrations, „β”—bending in-plane, „γ”—bending out-of-plane, „α(CCC)”—bending in-plane of the aromatic ring, ^b^ s—strong, m—medium, w—weak, v—very, sh—shoulder.

**Table 2:** The wavelengths of maximum absorbance λ_max_ [nm] from UV spectra of methanolic solutions of CA and its derivatives.

Compound	λ_max_ 1	λ_max_ 2	λ_max_ 3
CA	215	270	-
4-HCA	220	290	310
3,4-dHCA	215	290	320
3,4,5-tHCA	225	-	315

**Table 3:** Chemical shifts of CA; 4-HCA; 3,4-dHCA; and 3,4,5-tHCA in ^1^H and ^13^C NMR spectra [ppm]; atom numbering in Figure 3.

	**CA**		**4** **-** **HCA**		**3,4** **-** **dHCA**		**3,4,5** **-** **tHCA**	
**Proton Number**	**Exp.** [24]	**Theoret.**	**Exp.** [25]	**Theoret.**	**Exp.** [26,27]	**Theoret.**	**Exp.**	**Theoret.**
1	12.35	6.05	12.13	5.90	12.12	5.90	12.13	5.95
2	6.53	6.55	6.29	6.55	6.16	6.42	6.10	6.49
3	7.68	8.20	7.52	8.08	7.41	8.00	7.34	7.90
4	7.56	7.73	7.49	8.12	7.02	7.43	6.57	7.27
5	7.40	7.77	6.79	7.02	9.13	4.61	8.76	5.37
6	7.47	7.81	9.96	5.07	9.54	4.93	9.12	5.62
7	7.40	7.74	6.79	7.17	6.75	6.91	8.76	4.66
8	7.56	8.26	7.49	7.61	6.96	7.10	6.57	6.65
**Carbon Number**								
1	134.24	141.44	125.36	133.73	125.71	134.45	124.60	133.17
2	128.19	141.18	130.17	135.29	115.76	117.68	107.45	110.97
3	128.91	136.40	115.83	121.42	114.59	153.22	145.08	153.23
4	130.21	139.82	159.67	169.03	148.14	157.05	136.09	142.65
5	128.91	135.97	115.83	121.51	114.65	121.53	145.08	150.40
6	128.19	132.89	130.17	143.60	121.16	134.80	115.12	117.92
7	167.58	175.12	168.06	175.75	167.91	175.64	167.85	175.59
8	119.24	114.91	115.41	115.54	115.13	115.11	115.12	116.87
9	143.93	158.61	144.27	158.91	145.57	159.02	146.09	158.66

**Table 4:** Energy parameters and aromaticity indices for CA; 4-HCA; 3,4-dHCA; and 3,4,5-tHCA calculated in B3LYP/6-311++(d,p). Atoms numbering in Figure 3.

Parameter	CA	4-HCA	3,4-dHCA	3,4,5-tHCA
E_total_ [Hartree]	−498.371	−573.620	−648.862	−724.117
Energy [eV]	−13,561.3	−15,487.7	−17,656.4	−19,704.2
Dipole moment [Debye]	3.350	3.723	1.977	3.477
HOMA	0.968	0.962	0.949	0.967
GEO	0.011	0.018	0.025	0.014
EN	0.020	0.020	0.026	0.018
I6	94.43	93.07	91.78	93.72
Aj	0.995	0.992	0.989	0.994
BAC	0.916	0.886	0.850	0.898
NICS	−7.033	−8.008	−9.265	−10.711
sEDA	reference molecule	−0.135	−0.273	−0.228
pEDA	reference molecule	−0.199	−0.551	−0.836
HOMO [eV]	−9.796	−9.189	−8.848	−8.680
LUMO [eV]	−6.369	−6.127	−6.119	−6.111
Energy gap [eV]	3.427	3.063	2.729	2.569
Ionization energy I = −E_HOMO_ [eV]	9.796	9.189	8.848	8.680
Electron Affinity A = −E_LUMO_ [eV]	6.369	6.127	6.119	6.111
Electronegativity $\chi =\frac{I+A}{2}$ [eV]	8.082	7.658	7.484	7.396
Chemical potential $\mu =-\frac{I+A}{2}$ [eV]	−8.082	−7.658	−7.484	−7.396
Chemical hardness $\eta =\frac{I-A}{2}$ [eV]	1.714	1.531	1.365	1.285
Chemical softness $S=\frac{1}{2\eta }$ [eV]	0.292	0.327	0.366	0.389
Electrophilicity index $\omega =\frac{\mu^{2}}{2\eta }$ [eV]	19.060	19.148	20.523	21.287

**Table 5:** Cell growth of the *S. cerevisiae* KKP 512 in the culture medium at 28 °C after 24 h and 48 h of incubation in the presence of varied concentrations of phenolic acids: CA; 4-HCA; 3,4-dHCA; and 3,4,5-tHCA, with or without of hydrogen peroxide (H_2_O_2_). Initial yeast concentration (5.0 log CFU·mL^−1^).

	Cell Growth [log CFU·mL^−1^]						
	24 h				48 h		
	CA	4-HCA	3,4-dHCA	3,4,5-tHCA	4-HCA	3,4-dHCA	3,4,5-tHCA
15 mM	n.g.	n.g.	8.0 ± 0.2 ^b^	8.1 ± 0.1 ^b^	n.g.	7.6 ± 02 ^b^	7.7 ± 0.2 ^b^
7.5 mM	n.g.	7.4 ± 0.15 ^a^	7.9 ± 0.2 ^b^	7.6 ± 0.1 ^b^	7.4 ± 0.2 ^a^	7.7 ± 0.2 ^b^	7.8 ±0.01 ^b^
3 mM	n.g.	7.2 ± 0.2 ^a^	7.7 ± 0.1 ^b^	7.8 ± 0.2 ^b^	7.8 ± 0.2 ^b^	7.7 ± 0.1 ^b^	8.0 ± 0.1 ^b^
*S. cerevisiae* KKP 512	7.8 ± 0.05 ^b^				7.8 ± 0.05 ^b^		
	+4 mM H_2_O_2_						
15 mM	n.g.	n.g.	4.6 ± 0.3 ^a^	4.5 ± 0.1 ^a^	n.g.	7.6 ± 0.03 ^b^	7.8 ±0.02 ^b^
7.5 mM	n.g.	5.4 ± 0.1 ^b^	5.8 ± 0.3 ^b^	5.8 ± 0.1 ^b^	7.3 ± 0.5 ^a^	7.6 ± 0.1 ^b^	7.6 ± 0.3 ^b^
3 mM	n.g.	4.5 ± 0.3 ^a^	5.3 ± 0.1 ^b^	4.6 ± 0.1 ^a^	7.7 ± 0.1 ^b^	7.9 ± 0.06 ^b^	7.8 ± 0.2 ^b^
*S. cerevisiae* KKP 512	5.3 ± 0.5 ^b^				7.9 ± 0.06 ^b^		
	+2 mM H_2_O_2_						
15 mM	n.g.	n.g.	7.6 ± 0.12 ^b^	7.6 ± 0.1 ^b^	n.g.	7.7 ± 0.1 ^b^	7.7 ± 0.1 ^b^
7.5 mM	n.g.	7.1 ± 0.3 ^a^	7.7 ± 0.01 ^b^	7.6 ± 0.2 ^b^	7.1 ± 0.1 ^a^	7.7 ± 0.1 ^b^	7.8 ± 0.3 ^b^
3 mM	n.g.	7.2 ± 0.2 ^a^	7.0 ± 0.01 ^a^	7.4 ±0.02 ^a^	7.6 ± 0.2 ^b^	7.9 ± 0.2 ^b^	8.0 ± 0.2 ^b^
*S. cerevisiae* KKP 512	7.1 ± 0.4 ^a^				7.9 ± 0.01 ^b^		

n.g.—no growth (no single colony was found on the plate); means that not share the same letter are significantly different at *p* < 0.05.

## Data Availability

The data presented in this study are available on request from the corresponding author.

## Supplementary Materials

The following are available online at https://www.mdpi.com/article/10.3390/ma14206098/s1, Figure S1: FT-IR spectra of CA; 4-HCA; 3,4-dHCA; and 3,4,5-tHCA, Figure S2: Atom numbering of cinnamic acid and its derivatives (structures optimized in B3LYP/6-311**(d,p) level), Table S1: Geometrical parameters for CA; 4-HCA; 3,4-dHCA; and 3,4,5-tHCA calculated in B3LYP/6-311^++^(d,p). Atoms numbering in Figure S2, Table S2: The atomic charges (NBO and ESP) for CA; 4-HCA; 3,4-dHCA; and 3,4,5-tHCA calculated in B3LYP/6-311^++^(d.p). Atoms numbering in Figure S2, Table S3: Summary of the results concerning the biological activity of the tested compounds.
