# Supplementary material for: Natural Cinnamic Acid Derivatives: A Comprehensive Study on Structural, Anti/Pro-Oxidant, and Environmental Impacts
Source: Materials (Basel). 2021 Oct 15;14(20):6098. doi: 10.3390/ma14206098 (PMC8537339; doi:10.3390/ma14206098)
Supplement: Supplementary file 1 [file materials-14-06098-s001.zip › materials-1386523-supplementary.pdf]

Supplementary Materials

Natural Cinnamic Acid Derivatives: A Comprehensive Study on Structural, Anti/Pro-Oxidant, and Environmental Impacts

Kamila Gryko [1], Monika Kalinowska [1,*], Piotr Ofman [2], Renata Choińska [3], Grzegorz Świderski [1], Renata Świsłocka [1] and Włodzimierz Lewandowski [1]

[1] Department of Chemistry, Biology and Biotechnology, Faculty of Civil Engineering and Environmental Sciences, Białystok University of Technology, Wiejska 45E, 15-351 Białystok, Poland; k.gryko@pb.edu.pl (K.G.); g.swiderski@pb.edu.pl (G.Ś.); r.swislocka@pb.edu.pl (R.Ś.); w-lewando@wp.pl (W.L.)

[2] Department of Environmental Engineering Technology, Faculty of Civil Engineering and Environmental Sciences, Bialystok University of Technology, Wiejska 45E, 15-351 Białystok, Poland; p.ofman@pb.edu.pl

[3] Prof. Wacław Dąbrowski Institute of Agricultural and Food Biotechnology—State Research Institute, Rakowiecka 36, 02-532 Warsaw, Poland; renata.choinska@ibprs.pl

* Correspondence: m.kalinowska@pb.edu.pl

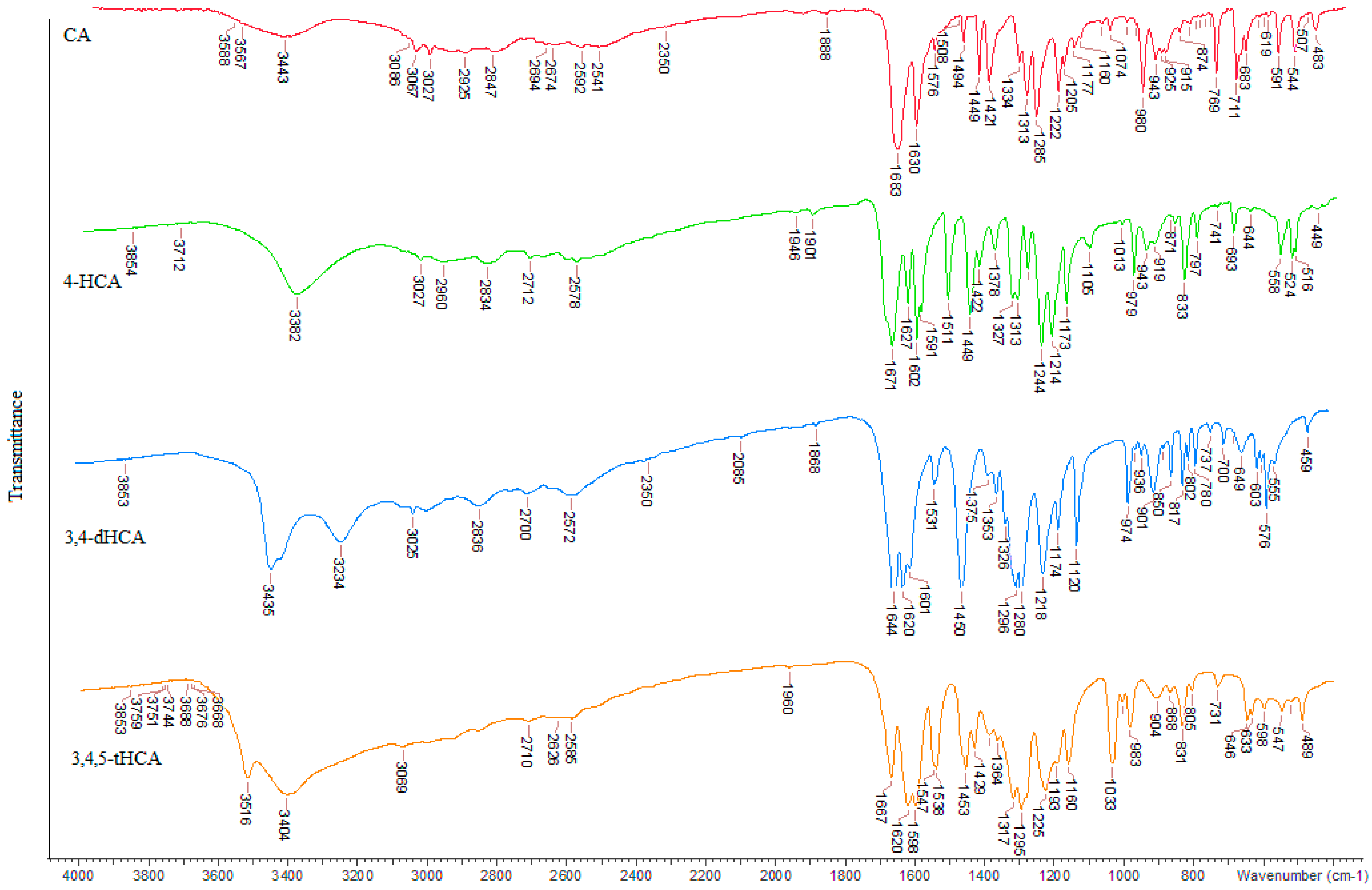

Figure S1. FT-IR spectra of CA, 4-HCA, 3,4-dHCA and 3,4,5-tHCA.

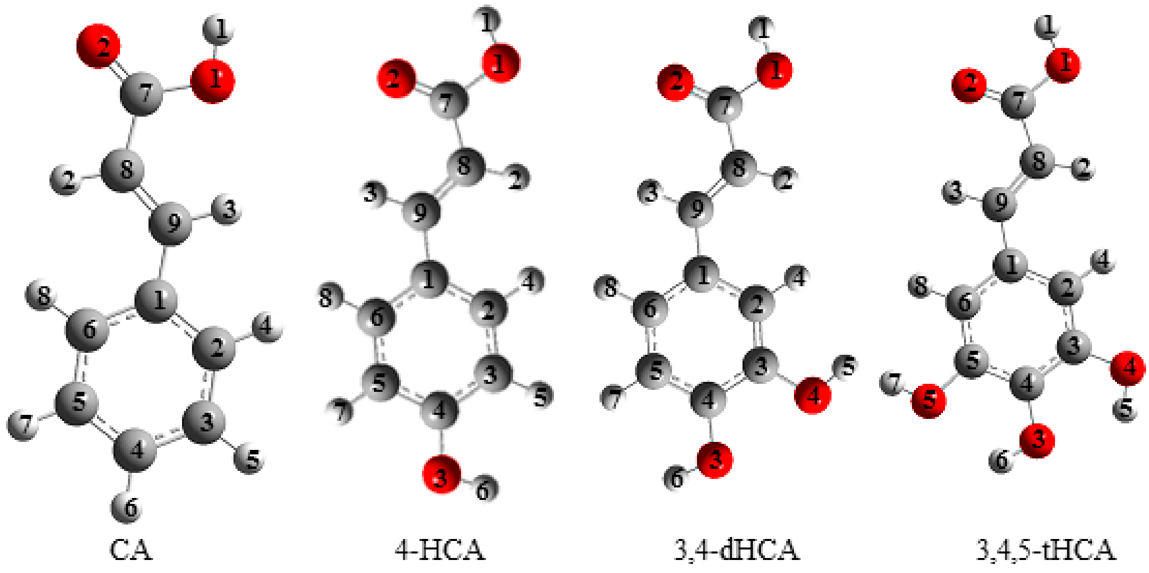

Figure S2. Atom numbering of cinnamic acid and its derivatives (structures optimized in B3LYP/6-311**(d,p) level).

Table S1. Geometrical parameters for CA, 4-HCA, 3,4-dHCA and 3,4,5-tHCA calculated in B3LYP/6-311^{++}(d,p). Atoms numbering in **Figure S2**.

	**CA**	**4-hCA**	**3,4-dHCA**	**3,4,5-tHCA**
Distances				
C1-C2	1.40462	1.40583	1.40895	1.40383
C2-C3	1.39178	1.38618	1.38447	1.38721
C3-C4	1.39287	1.40005	1.41139	1.40005
C4-C5	1.39714	1.39552	1.39115	1.39312
C5-C6	1.38831	1.38673	1.39163	1.38794
C6-C1	1.40637	1.40683	1.40057	1.40649
C1-C9	1.46355	1.45731	1.45640	1.45889
C7-C8	1.46891	1.46948	1.46932	1.47036
C8-C9	1.34408	1.34505	1.34519	1.34418
C3-O4	-	-	1.36339	1.36054
C4-O3	-	1.36326	1.35917	1.36641
C5-O5	-	-	-	1.37578
C7-O1	1.36239	1.36333	1.36333	1.36211
C7-O2	1.21160	1.21162	1.21163	1.21183
O1-H1	0.96791	0.96840	0.96843	0.96843
O3-H6	-	0.96326	0.96331	0.96610
O4-H5	-	-	0.96304	0.96621
O5-H7	-	-	-	0.96237
C2-H4	1.08495	1.08338	1.08540	1.08191
C3-H5	1.08403	1.08585	-	-
C4-H6	1.08404	-	-	-
C5-H7	1.08411	1.08299	1.08589	-
C6-H8	1.08338	1.08474	1.08372	1.08523

C8-H2	1.08281	1.08275	1.08285	1.08256
C9-H3	1.08607	1.08767	1.08736	1.08747
Angles				
C2-C3-C4	119.947	120.026	120.759	120.055
C3-C4-C5	119.701	119.881	119.211	119.834
C4-C5-C6	120.341	119.509	119.633	120.370

Table S2. The atomic charges (NBO and ESP) for CA, 4-HCA, 3,4-dHCA and 3,4,5-tHCA calculated in B3LYP/6-311^{++}(d.p). Atoms numbering in **Figure S2.**

	**CA**		**4-HCA**		**3,4-dHCA**		**3,4,5-tHCA**	
Atom No.	NBO	ESP	NBO	ESP	NBO	ESP	NBO	ESP
C1	-0.096	1.269	-0.125	1.364	-0.107	0.278	-0.084	0.167
C2	-0.164	0.322	-0.142	-1.297	-0.233	-0.442	-0.228	-0.407
C3	-0.207	-0.366	-0.282	-0.130	0.282	0.368	0.294	0.429
C4	-0.185	-0.295	0.334	-0.523	0.297	0.227	0.224	0.020
C5	-0.196	-0.367	-0.241	-0.312	-0.268	-0.136	0.262	0.349
C6	-0.166	-1.194	-0.143	0.366	-0.166	-0.312	-0.254	-0.476
C7	0.758	0.054	0.758	0.040	0.761	0.751	0.761	0.731
C8	-0.294	-0.121	-0.307	-0.191	-0.321	-0.285	-0.312	-0.295
C9	-0.106	-0.237	-0.104	-0.126	-0.092	-0.064	-0.095	0.074
H1	0.485	0.291	0.484	0.292	0.482	0.374	0.482	0.374
H2	0.213	0.211	0.211	0.201	0.207	0.144	0.212	0.118
H3	0.215	0.194	0.214	0.171	0.218	0.062	0.215	0.024
H4	0.205	0.157	0.207	0.077	0.200	0.182	0.219	0.231
H5	0.209	0.170	0.205	0.159	0.469	0.390	0.487	0.384
H6	0.207	0.156	0.470	0.271	0.469	0.349	0.499	0.392
H7	0.208	0.191	0.222	0.192	0.205	0.107	0.483	0.388
H8	0.205	0.064	0.207	0.172	0.209	0.163	0.206	0.206
O1	-0.701	-0.209	-0.703	-0.185	-0.691	-0.602	-0.688	-0.588
O2	-0.596	-0.291	-0.607	-0.317	-0.615	-0.545	-0.617	-0.542
O3	-	-	-0.663	-0.224	-0.649	-0.457	-0.696	-0.474
O4	-	-	-	-	-0.657	-0.561	-0.667	-0.572
O5	-	-	-	-	-	-	-0.703	-0.541

Table S3. Summary of the results concerning the biological activity of the tested compounds.

Compound	DPPH• inhibition - IC_{50} [mM]	HO• inhibition (C=0.1mM) [%]	FRAP (C=0.05mM) [μM_{Fe2+}]	CUPRAC (C=0.05mM) [$\mu M_{troloxu}$]	Trolox pro-oxidation - 60 min (C=2.5; 5μM) [%]	Linoleic acid peroxidation inhibition - day 5. (C=5mM) [%]	E_{HOMO} [eV]	ΔE [eV]	Ionization potential [eV]
**CA**	> 6.0	48.40 ± 0.83	-	-	0.20 ±1.09; 4.35 ± 0.91	30.76 ± 1.22	-9.79556	3.427	9.79556
**4-HCA**	> 2.7	49.99 ± 0.58	0.74 ± 0.06	6.40 ± 0.55	27.63 ± 1.58; 70.57 ± 1.18	49.49 ± 2.18	-9.18928	3.063	9.18928
**3,4-dHCA**	0.011 ± 0.001	50.22 ± 0.55	15.45 ± 0.49	17.99 ± 0.94	134.78 ± 4.65; 230.28 ± 1.73	82.77 ± 0.12	-8.84833	2.729	8.84833
**3,4,5-tHCA**	0.004 ± 0.002	53.02 ± 0.80	29.56 ± 2.02	20.86 ± 0.81	17.15 ±0.96; 30.18 ± 0.48	88.01 ± 0.88	-8.68016	2.569	8.68016
